# Fat Imaging via Magnetic Resonance Imaging (MRI) in Young Children (Ages 1-4 Years) without Sedation

**DOI:** 10.1371/journal.pone.0149744

**Published:** 2016-02-22

**Authors:** Grace E. Shearrer, Benjamin T. House, Michelle C. Gallas, Jeffrey J. Luci, Jaimie N. Davis

**Affiliations:** 1 Nutritional Sciences, University of Texas, Austin, Texas, United States of America; 2 Department of Pediatrics, University of Texas Southwestern, Austin, Texas, United States of America; 3 Department of Neuroscience, University of Texas, Austin, Texas, United States of America; The Chinese University of Hong Kong, HONG KONG

## Abstract

**Introduction:**

This pilot study developed techniques to perform Magnetic Resonance Imaging (MRI) of specific fat deposition in 18 children (age 18 months to 4 years).

**Methods:**

The children engaged in a series of practice tests to become acclimated to the scanner noises, reduce claustrophobia, and rehearse holding still for a set time. The practice tests assessed if the child could remain still for two minutes while watching a video, first while lying on a blanket, second, on the blanket with headphones, and third, in the mock scanner. The children who passed the three practice tests were then scanned with a 3T Siemens Skyra magnet. Abdominal fat distribution (region of interest (ROI) from the top of the ileac crest to the bottom of the ribcage) volume was measured using 2-point DIXON technique. This region was chosen to give an indication of the body composition around the liver.

**Results:**

Twelve out of eighteen participants successfully completed the actual MRI scan. Chi-squared test showed no significant difference between male and female pass-fail rates. The median age of completed scans was 36 months, whereas the median age for children unable to complete a scan was 28 months. The average total trunk fat was 240.9±85.2mL and the average total VAT was 37.7±25.9mLand liver fat was not quantifiable due to physiological motion. Several strategies (modeling, videos, and incentives) were identified to improve pediatric imaging in different age ranges.

**Conclusion:**

Using an age-specific and tailored protocol, we were able to successfully use MRI for fat imaging in a majority of young children. Development of such protocols enables researchers to better understand the etiology of fat deposition in young children, which can be used to aid in the prevention and treatment of adiposity.

## Introduction

Overall prevalence of type 2 diabetes (T2D) has increased 30.5% from 2001 to 2009 in children ages 10–19 years [1]. Visceral adipose tissue, subcutaneous adipose tissue, and liver fat have emerged as important risk factors of T2D [2]. Imaging via magnetic resonance (MRI) is a noninvasive method for quantifying visceral and subcutaneous fat, as well as detection of fat deposits in the liver. While the cause of the increase in T2D in children is currently unknown, research targeted at younger ages is of increasing value in discovering the etiology of T2D in children.

The use of MRI is an increasingly preferred technique to measure visceral and liver fat depots in pediatric populations [3]. MRI has been documented as a reliable method for imaging fat free of inter-observation variation [4], with a high degree of correlation compared to histopathological results [5]. Unlike the computed tomography (CT) or dual energy absorptiometry (DXA), the use of MRI eliminates exposure to potentially harmful ionizing radiation [6], making MRI particularly attractive for research with children [7]. Additionally, children in this age group (6 to 48 months) are more susceptible to the detrimental effects of ionizing radiation [8].

Currently, research using MRI to quantify fat tissue in young children (6 to 48 months) is limited. A majority of the MRI research in young children is focused on brain imaging, with sleeping children [9–13]. Most children undergoing an abdominal MRI at a young age require sedation or anesthesia to minimize motion (both voluntary and physiologic) in the magnet [14]. Due to the risks and challenges associated with imaging anesthetized children [14], developing a non-sedated protocol for researchers is warranted. A protocol to image the abdominal cavity without sedation in young children would allow for earlier detection and study of fat deposits in high resolution. This would be beneficial for the study of not only T2D, but also “catch up” growth and obesity research, epigenetic programing in childhood, and may have diagnostic benefits for early detection of enlarged heart or other gross anatomical problems without the use of radiation. The purpose of this efficacy study is to develop and test a protocol to perform abdominal fat imaging in a small sample of children 12 to 48 months of age. This study hypothesizes that imaging to measure fat tissue is logistically feasible in young children (12–48 mo.) with an MRI scanning protocol designed and optimized for pediatric subjects.

## Materials and Methods

### Study participants

The Institutional Review Board at the University of Texas at Austin approved this pilot study. Participants were recruited primarily through word of mouth or email advertisements with local childcare centers and health clinics. Parents of children 12 to 48 months of age were invited to enroll their children to participate. Children with attention deficit hyperactivity disorder, a history of claustrophobia, or developmental delays such as Down’s syndrome were excluded from this study via a prescreening questionnaire [15]. Written informed consent was obtained from the participant’s parent or guardian before any testing. All consent forms were logged and are kept in a locked secure location.

### Description of the protocol

**Fig 1:**
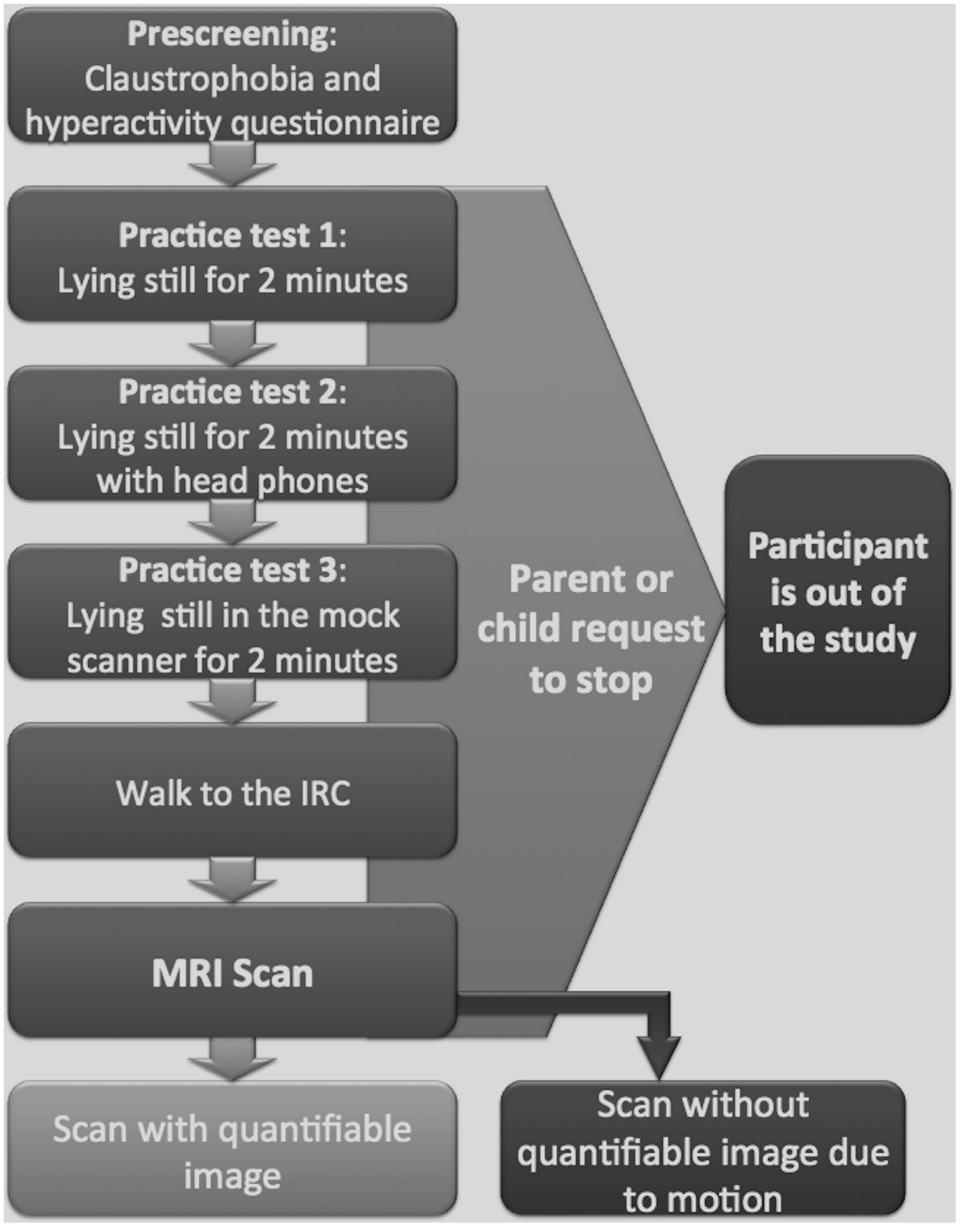
Outline of study flow. Children were given three attempts to complete each practice test. Children who failed practice tests 1 and 2 were given the option to try practice test 3.

A tailored protocol for imaging children 12 to 48 months of age was developed in accordance with current MRI best practices. The protocol involved three practice tests utilizing a simulated scanning environment (a mock MRI scanner) and a final MRI scan with positive reward-based reinforcement. Guardian/adult role modeling was encouraged throughout the procedure (Fig 1. Outline of study flow, children were given three attempts to complete each practice test. Children who failed practice tests 1 and 2 were given the option to try practice test 3).

Initially, a series of questions were asked of the guardian in order to determine whether or not the child had any MR contradictions. Given that children are at risk for ingesting small objects or putting small objects in their ears, nose, mouth, the child was then examined with a Garrett Super Scanner V (Garrett Electronics, Inc Garland, TX) hand-held metal detector to ensure that the he or she had not ingested or hidden any metal or magnetic objects. This step was best performed with the child standing on a plastic step stool, as any metal elements in the floor sporadically resulted in the hand-held metal detector buzzing and giving a false positive. The guardian was then asked a series of demographic questions addressing socioeconomic status and ethnicity. Next, the child selected a movie to watch from variety age-appropriate movies. The child’s height and weight were measured to the nearest 0.1 kg and 0.1 cm using a Seca scale and stadiometer (SECA 270, 220 Chino, CA). Before each practice test, the child was shown a bin of age appropriate toys to incentivize minimum motion. The toys bins increased in desirability with the difficulty of the test. Throughout each test, positive encouragement was used to promote calm and minimize anxiety for the child.

The first practice test was used to determine if the child could lie still for 2 minutes. Two minutes of imaging was the critical period for the child to remain motionless. Researchers laid a brightly-colored blanket down on the floor and instructed the child to lie down. If the child was shy or did not understand the instructions the researcher or guardian modeled the behavior. The guardian was also encouraged to lie next to the child during this test. While the child was lying still on the blanket, the movie of his/her choice was shown via an iPad (Apple Inc, Cupertino, CA) held above the child by a researcher and a timer was started. If the child moved during the test, the movie was paused and the timer was restarted. The child was verbally instructed to remain motionless, and was allowed three opportunities to complete the test.

The second practice test was designed to determine whether the child could remain still while wearing headphones and have a heavy blanket draped over his/her stomach to acclimate the child to wearing headphones and to simulate the pediatric abdominal RF coils needed for the actual scan. The child was instructed to lie down on the blanket on the floor, as in practice test one, however this time the child was outfitted with child-sized headphones playing the audio from the chosen movie. The child was instructed to remain still and timed for two minutes, while they watched the movie as in practice test one. Again, the movie was played via the iPad and was paused if the child moved. If necessary, the researcher or guardian also modeled lying still with the headphones. If the child was not visibly distraught, he or she was allowed to attempt the third test. Even if the child was unable to complete practice tests one and two, he or she was allowed to do practice test three. This was allowed as most children were curious about the mock scanner and were interested in test three.

The third practice test was designed to determine whether the child could remain still in the mock scanner. The mock scanner was the shell of a General Electric Excite HD 3T MRI scanner with the operational components removed; its sole purpose was to simulate an MRI-like environment. The scanner was described as a big “donut” or “spaceship”, and child was informed that the MRI makes big noises like a, “choo choo train.” The mock scanner was equipped with a monitor and mirror to allow the child to continue watching his/her movie once inside the mock scanner. A novel mirror was designed specially to work without the use of a head coil, which is normally the only time a mirror would be used in an MRI. The mirror was supported on a flexible, articulated plastic neck (½” diameter Loc-Line, Lockwood Products, Lake Oswego, OR) which attached to the scanner bed via a polycarbonate bracket and heavy duty Velcro strips, which allowed for adjustment of the mirror for optimal viewing of the movie for the child. The movie was projected behind the child flipped left to right in order to allow the child to watch it via the mirror. The mock scanner was also equipped with speakers, through which a recording of the actual MRI noises was played. A researcher or the guardian would first model lying still and being slid into the scanner. Researchers encouraged the child to help slide the researcher or their guardian into the scanner, and described the scanner as a “ride.” The researcher or guardian always modeled going into the bore of the mock scanner as fun and exciting, and then remained still in the scanner and watched the movie. The researchers and guardians encouraged motionlessness in the scanner through positive encouragement to the model (in the same manner as they would the child).

The child was placed on the mock scanner bed and outfitted with the appropriate-sized headphones, through which the sounds of the movie were played. After being pushed smoothly into the bore, the child was instructed to remain still and to continue watching his/her movie. Once the child was on the scanner bed and slid into the bore, the guardian was advised to stand at the front of the scanner near the child’s head and in their field of view. The two-minute timer and movie was started, and if the child moved the movie was stopped, the timer restarted, and the child was encouraged to remain still. The child was allowed three attempts to complete the mock scan. Only upon completion of the mock scan, the child was allowed proceed to the real MRI scan.

After the three practice tests, the child, guardian, and research team walked to the Imaging Research Center (IRC) where the actual MRI scan would take place. The walk to the IRC lasted about five minutes and allowed for the child to dissipate excess energy outside of the research environment. The child was given a snack break, after which child and guardian were once again checked for metal objects. After the snack, the child and guardian were encouraged to enter the MRI room and become acclimated to the environment and machine. The child was reassured that this machine was just a fancier version of the previous “donut” or “spaceship.” The child was shown the final prize bin and was reminded that they would receive the final prize after the real scan. The child was instructed to lie still on the bed and was fitted with headphones and a size-matched flexible coil array. The guardian and one researcher sat at the head of the magnet, visible to the child. Encouraging the guardian to stand at the head of the scanner allowed both the child and guardian to be in physical contact. The bed of the scanner was equipped with a mirror and projector, similar to the mock scanner described above, so the child could watch the movie while in the bore of the magnet. The child was allowed three attempts to complete the imaging. The scan was deemed a success if the image quality was sufficient to gather necessary data as assessed by the research team (i.e. little ghosting, few artifacts) described in the fat mass quantification section bellow. Table 1 outlines best practices used throughout the study.

**Table 1:** Challenges and solutions in pediatric research MRI.

Complication	Solution
Metal objects	Full body examination with highly sensitive metal detecting wand; Used metal detector while the child was standing on a plastic step stool, to avoid interference from the floor; Set the metal detector wand to vibrate to avoid frightening the child or parent; Modeled the examination on the guardian or fellow researcher first
Hyperactivity	The hyperactivity questionnaire screened out any participants with diagnosed hyperactivity; A walking break in between the mock scanner and the MRI allowed the child to expend energy
Anxiety from loud noises	Child sized earplugs reduced noise for children who were not compliant with headphones; Introducing the MRI noises in the mock scanner desensitized the child and parent to the scanner noises and volume; The mock scanner noises were faded in, allowing the child to acclimate
Anxiety about the MRI	The mock scanner allowed the child to experience the MRI and become accustomed to the bore of the magnet and the noises; The child was thoroughly debriefed before any testing and was repeatedly asked if he or she had any questions or were scared.
Stillness	A series of tests and subsequent rewards; The movie was paused when the child moved to remind them to be still
Claustrophobia	The mock scanner allowed for screening of children with latent claustrophobia; Practice in the mock scanner allowed the child become familiar with the scanning process; Encouraging the parent to stand at the head of the scanner and touch the child alleviated stress for both the parent and child
Shyness/ stranger anxiety	Designated a leader who predominantly interacts with the child; Female children tended to prefer a female designated leader; The parent was involved in the entire process to alleviate shyness an anxiety.

### Image Acquisition

**Fig 2:**
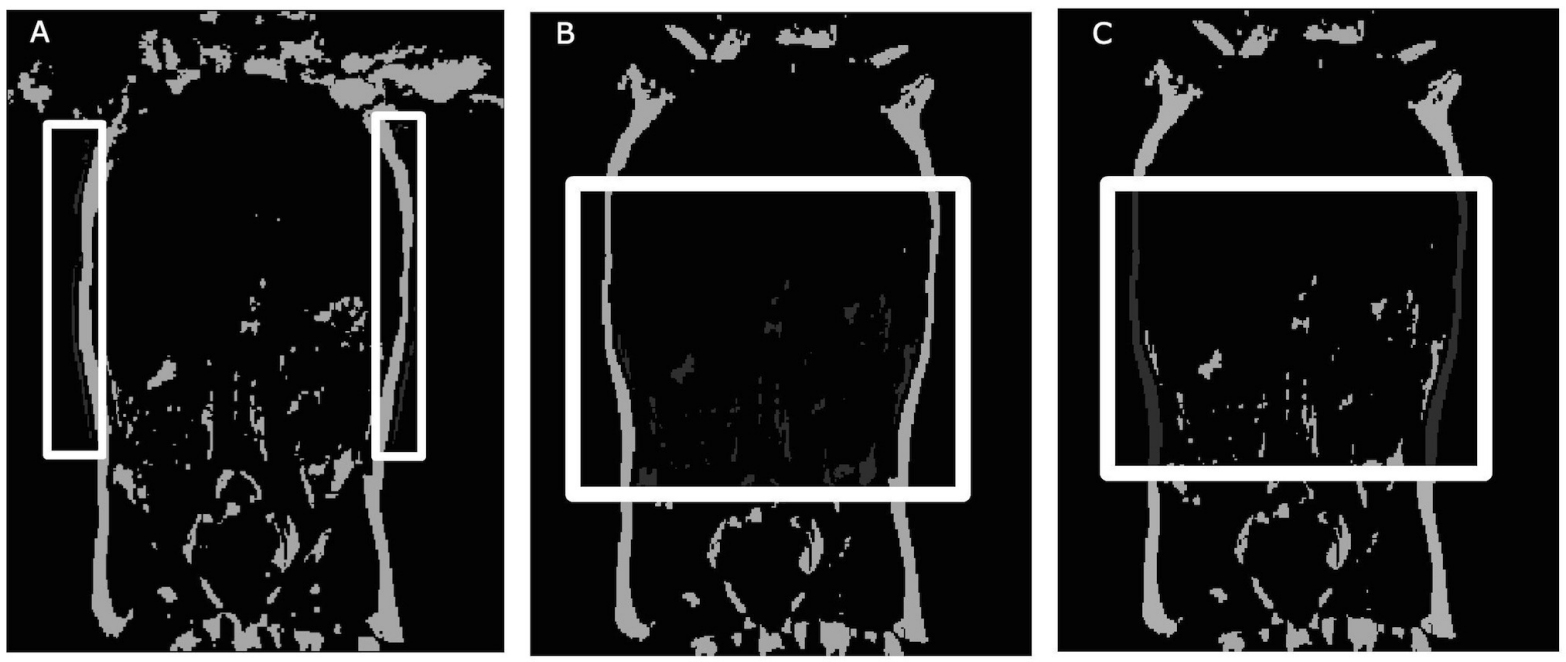
Images representing unusable and usable (both visceral adipose (VAT) and subcutaneous adipose tissue (SAT)). A) Unusable image due to ghosting, highlighted in the white boxes. B) Usable image with VAT represented in dark gray. The white box represents the region of interest. C) Usable image with SAT represented in dark gray. The white box represents the region of interest.

Visceral adipose tissue (VAT), subcutaneous adipose tissue (SAT), and liver fat volume were measured using a vibe 2-point DIXON technique with Cartesian sampling. This scanning protocol contained one slab with 44 slices, each 3mm thick to allow for imaging of the entire abdominal area in the coronal direction. The field of view (FoV) was 380mm, and the phase FoV was 78.1%, with a repetition time (TR) of 4.58 seconds, an in-phase echo time (TE) of 2.56ms, and an out-phase TE of 1.34ms, and a flip angle of 9.0 degrees. One set each of in-phase images and one out-of-phase images were acquired [6]. Image resolution was 1.1875 mm^2^ in both directions, and the image matrix was. The time in the scanner lasted 2.5 minutes, with each VIBE sequence lasting 17 s. The Siemens Skyra 3T used the 32-channel coil array integrated into the patient bed/table. Depending on the child’s size, a 4-channel large array coil or a 4-channel small array coil was placed anteriorly and used in combination with the 32-channel coil to provide full abdominal coverage. The number of coil elements used with the 32 channel elements also depended on the child’s size; the maximum coils used were 17, the minimum were 8, and the average coil elements used were 12. Using a high number of coil elements in this way made an ideal experimental configuration to take advantage of a partially parallel image acquisition acceleration method. The GeneRalized Auto-calibrating Partially Parallel Acquisitions (GRAPPA) technique used for this study to accelerate image acquisition to make the scan short enough for the challenging parameters of this study, an acceleration factor of two was used. Due to the young age of the subjects, a breath hold was not feasible and therefore was not used. Fat volume fraction and fat mass fraction were computed on a voxel-by-voxel basis, and averaged over each segmented organ. VAT and SAT were measured as a region of interest (ROI) from the top of the ileac crest to the top of the ribcage (Fig 2). The benefits of using ROI for liver fat and body fat quantification as well as challenges associated with fat quantification from MRI can be found here [16]. This region was chosen to give an indication of the body composition around the liver.

### Fat mass quantification

Percent water and fat were calculated using a novel quantification program developed in house based on the Otsu method [17] and run in MATLAB (version R2013a, MathWorks Inc, Natick, MA). Initially, the total body fat was calculated and the subcutaneous fat was then calculated. The removal of the SAT from the total body fat yielded VAT. All fat measurements are reported as a proportion to control for differences in body size among the subjects. Liver fat quantification was attempted, however physiologic motion, due to free breathing, impaired quantification.

### Statistical Analysis

The MRI was considered a success if the scan yielded at least 50% quantifiable images from the 44 slices acquired. The cut off of 50% was determined as of 44 slices, a minimum of 22 slices characterized the liver environment (the abdominal cavity where the liver is visible). This was deemed as the most important as visceral fat surrounding the organs appears to be partially detrimental [18]. Non-quantifiable images were defined as exhibiting high ghosting and inability to define the ileac crest and or bottom of the rib cage (fig 2). Chi squared tests were run to determine differences between pass-fail rates between age groups (children 12 to 35 months and 36 to 42 months) and sex.

## Results

Demographics are shown in Table 2. Chi-squared test showed no significant difference between male and female pass-fail rates. The youngest completed and readable scan was obtained from a subject at 17 months of age. Additionally, Chi-squared test revealed no difference between pass-fail rates of children 12 to 35 months and 36 to 42 months.

**Table 2:** Demographics and Fat quantification by age group.

	Total	12–35 Months	36–48 months
**Entered in study**	18	9	9
*Sex (female)*	10	7	3
*Age (months)*	33.2±12.9	23.3±4.7	44.2±7.3
*Height (cm)*	96.3±10.6	89.9±9.0	103.4±7.5
*Weight (kg)*	14.6±3.7	12.9±4.2	16.3±1.9
*BMI percentile*	36.6±32.1	32.6±30.6	40.5±38.2
**Readable scan**	12	5	7
*Sex (female)*	7	5	2
*Age (months)*	36.0±12.0	25.9±6.7	44.7±7.8
*Height (cm)*	99.0±9.3	93.3±7.5	103.9±6.7
*Weight (kg)*	15.5±3.5	14.2±4.6	16.6±2.1
*BMI percentile*	37.2	35.2±35.9	38.93±40.1
Trunk fat total (ml)	240.9±85.2	238.9±85.7	243.1±84.2
Subcutaneous trunk fat (ml)	129.2±51.5	121.6±39.3	135.7±62.5
Visceral trunk fat (ml)	37.7±25.9	44.9±27.3	31.2±24.8

All data presented as mean ± standard deviation except entered in study, readable scan, and sex which are presented as a counts.

Table 2 also displays the mean fat depots by age range for participants who completed the scan. While VAT and SAT fat was quantifiable, the small sample size precluded statistical analysis between age and sex groups, therefore the descriptives are presented. Of note, without a breath hold, physiological motion (breathing) consistently over estimated liver fat quantification due to bleeding of water and fat voxel intensities. Therefore, liver fat measures are not included in the table.

## Discussion

The purpose of this study was to examine the feasibility of using MRI to assess adipose distribution in children ages 12 to 48 months. This study found that using MRI to measure VAT and SAT is feasible in children 12 to 48 months. Expansion of these techniques to include liver fat would necessitate respiratory gating. Demographics and fat quantification can be found in Table 2. Pediatric MR imaging with these techniques is possible in children as young as 17 months under certain conditions, which is a significant improvement upon the status quo considering the aforementioned lack of any MRI scanning data in children 6 months to 5 years of age. Table 3 outlines the researchers’ observations regarding the specific age groups and which methods were more effective for each group.

**Table 3:** Effective MRI preparation strategies by age group.

Age Group	Effective strategies
12–35 Months	Modeling and instruction with a puppet; Receptive to guardian modeling the instructions, such as lying on the ground or in the mock scanner; Had difficulty wearing headphones, but perform well with child sized ear plugs
36–48 months	Using toy reward bins as incentive for stillness and task completion; Receptive to verbal instructions from a researcher at their eye level; Receptive to one lead researcher and a guardian leading him or her through tasks; Female children were more receptive to a female researcher; Modeling the behavior was effective; Accepting to wearing headphones

This study identified successful strategies that enabled acquisition of the scans that worked in each age/sex group. The imaging protocol that we designed addressed many of the documented problems of pediatric imaging including: claustrophobia, anxiety (due to a medical procedure or noise), hyperactivity, and lack of patient knowledge [19]. The use of reward-based learning was particularly successful. The children were much more likely to remain still if they knew they could get a reward. Furthermore, a key to a successful scan was a supportive guardian. Positive interactions between the guardian, designated leader, and child was crucial for a quality scan. These interactions were enhanced if the guardian was actively involved in the protocol, and had a positive attitude. The effect of an anxious guardian on the MRI procedure has been noted before [20]; it was critical to keep the guardian and the child engaged to prevent anxiety. The method of acclimating the child to the MRI environment through practice tests and a mock MRI was essential to a successful scan. The benefits of the mock MRI have been shown repeatedly [21]. The ability to pass practice test three but not the first two could be due to the mock scanner itself making the test more interesting to the child. The mock scanner was also presented as a ride and therefore was more enjoyable than the practice tests on the blanket on the floor. The mock scanner appears to be the most useful for training for the researcher, and the most interesting to the participant, and may therefore be sufficient for most children.

The method of quantification in this study relied heavily on the operator. The operator must have prerequisite knowledge of abdominal anatomy. However, the quantification of 20 to 25 slices increased accuracy and allowed for measurement of fat in three dimensions, rather than using area from one slice at the lumbar region [22–27].

Without a breath hold or prospective respiratory gating, imaging hepatic fat in this population is not currently feasible. Further work is needed to develop imaging and liver fat qualification with and without a breath hold in young children. Another limitation of this study is the small sample size. Additionally, this protocol requires the use of trained staff that work well with children. Nevertheless, the protocol described is an effective method to control for anxiety in the child and guardian, while promoting desired behaviors such as stillness. This protocol could conceivably also be tailored to older pediatric groups as well.

Given the increase in pediatric T2D incidence, early childhood imaging is critical in understanding etiology of these diseases. The ability to quantify visceral and subcutaneous fat in a very young population is a useful tool for intervention studies to quantify when and where fat is accumulated in early life.

## References

[1] Dabelea D, Mayer-Davis EJ, Saydah S, Imperatore G, Linder B, Divers J, et al Prevalence of type 1 and type 2 diabetes among children and adolescents from 2001 to 2009. JAMA. American Medical Association; 2014;311: 1778–86. 10.1001/jama.2014.3201 PMC4368900 24794371

[2] Gastaldelli A. Abdominal fat: does it predict the development of type 2 diabetes? Am J Clin Nutr. 2008;87: 1118–1119. Available: http://ajcn.nutrition.org/content/87/5/1118.full 18469227 10.1093/ajcn/87.5.1118

[3] Schwimmer JB, Celedon MA, Lavine JE, Salem R, Campbell N, Schork NJ, et al Heritability of Nonalcoholic Fatty Liver Disease. Gastroenterology. 2009;136: 1585–1592. 10.1053/j.gastro.2009.01.050 19208353 PMC3397140

[4] Vajro P, Lenta S, Socha P, Dhawan A, McKiernan P, Baumann U, et al Diagnosis of nonalcoholic fatty liver disease in children and adolescents: position paper of the ESPGHAN Hepatology Committee. J Pediatr Gastroenterol Nutr. 2012;54: 700–13. 10.1097/MPG.0b013e318252a13f 22395188

[5] Kim SH, Lee JM, Han JK, Lee JY, Lee KH, Han CJ, et al Hepatic macrosteatosis: predicting appropriateness of liver donation by using MR imaging—correlation with histopathologic findings. Radiology. 2006;240: 116–129. 10.1148/radiol.2393042218 16684918

[6] Hu HH, Kim H-W, Nayak KS, Goran MI. Comparison of fat-water MRI and single-voxel MRS in the assessment of hepatic and pancreatic fat fractions in humans. Obesity (Silver Spring). 2010;18: 841–847. 10.1038/oby.2009.352 19834463 PMC2847037

[7] Armpilia CI, Fife IAJ, Croasdale PL. Radiation dose quantities and risk in neonates in a special care baby unit. Br J Radiol. 2002;75: 590–595. 12145132 10.1259/bjr.75.895.750590

[8] Brody AS, Frush DP, Huda W, Brent RL. Radiation risk to children from computed tomography. Pediatrics. 2007;120: 677–82. 10.1542/peds.2007-1910 17766543

[9] Deoni SCL, Dean DC, Piryatinsky I, O’Muircheartaigh J, Waskiewicz N, Lehman K, et al Breastfeeding and early white matter development: A cross-sectional study. Neuroimage. 2013;82: 77–86. 10.1016/j.neuroimage.2013.05.090 23721722 PMC3777218

[10] Dean DC, Dirks H, O’Muircheartaigh J, Walker L, Jerskey BA, Lehman K, et al Pediatric neuroimaging using magnetic resonance imaging during non-sedated sleep. Pediatr Radiol. 2014;44: 64–72. 10.1007/s00247-013-2752-8 23917588 PMC3889986

[11] Dean DC, O’Muircheartaigh J, Dirks H, Waskiewicz N, Lehman K, Walker L, et al Modeling healthy male white matter and myelin development: 3 through 60months of age. Neuroimage. 2014;84: 742–52. 10.1016/j.neuroimage.2013.09.058 24095814 PMC3895775

[12] Wolff JJ, Gu H, Gerig G, Elison JT, Styner M, Gouttard S, et al Differences in white matter fiber tract development present from 6 to 24 months in infants with autism. Am J Psychiatry. 2012;169: 589–600. 10.1176/appi.ajp.2011.11091447 22362397 PMC3377782

[13] Almli CR, Rivkin MJ, McKinstry RC. The NIH MRI study of normal brain development (Objective-2): newborns, infants, toddlers, and preschoolers. Neuroimage. 2007;35: 308–25. 10.1016/j.neuroimage.2006.08.058 17239623

[14] Serafini G, Zadra N. Anaesthesia for MRI in the paediatric patient. Curr Opin Anaesthesiol. 2008;21: 499–503. 10.1097/ACO.0b013e328304115b 18660661

[15] Valentiner DP, Telch MJ, Petruzzi DC, Bolte MC. Cognitive mechanisms in claustrophobia: An examination of reiss and McNally’s expectancy model and Bandura's self-efficacy theory. Cognit Ther Res. 1996;20: 593–612. 10.1007/BF02227963

[16] Hu HH, Nayak KS, Goran MI. Assessment of abdominal adipose tissue and organ fat content by magnetic resonance imaging. Obes Rev. 2011;12: e504–15. 10.1111/j.1467-789X.2010.00824.x 21348916 PMC3079791

[17] Otsu N. A Threshold Selection Method from Gray-Level Histograms. Automatica. 1975;11: 23–27. Available: http://www.researchgate.net/publication/202972390_A_Threshold_Selection_Method_from_Gray-Level_Histograms

[18] Goran MI, Gower BA. Relation between visceral fat and disease risk in children and adolescents. Am J Clin Nutr. 1999;70: 149S–56S. Available: http://www.ncbi.nlm.nih.gov/pubmed/10419419 10.1093/ajcn/70.1.149s 10419419

[19] Hallowell LM, Stewart SE, de Amorim e Silva CT, Ditchfield MR. Reviewing the process of preparing children for MRI. Pediatric Radiology. 2008 pp. 271–279. 10.1007/s00247-007-0704-x 18084752

[20] Raschle N, Zuk J, Ortiz-Mantilla S, Sliva DD, Franceschi A, Grant PE, et al Pediatric neuroimaging in early childhood and infancy: Challenges and practical guidelines. Ann N Y Acad Sci. 2012;1252: 43–50. 10.1111/j.1749-6632.2012.06457.x 22524338 PMC3499030

[21] De Amorim E Silva CJT, Mackenzie A, Hallowell LM, Stewart SE, Ditchfield MR. Practice MRI: Reducing the need for sedation and general anaesthesia in children undergoing MRI. Australasian Radiology. 2006 pp. 319–323. 10.1111/j.1440-1673.2006.01590.x 16884416

[22] Fox KR, Peters DM, Sharpe P, Bell M. Assessment of abdominal fat development in young adolescents using magnetic resonance imaging. Int J Obes Relat Metab Disord. 2000;24: 1653–9. Available: http://www.ncbi.nlm.nih.gov/pubmed/11126220 11126220 10.1038/sj.ijo.0801464

[23] Fox K, Peters D, Armstrong N, Sharpe P, Bell M. Abdominal fat deposition in 11-year-old children. Int J Obes Relat Metab Disord. 1993;17: 11–6. Available: http://www.ncbi.nlm.nih.gov/pubmed/8383635 8383635

[24] Owens S, Gutin B, Barbeau P, Litaker M, Allison J, Humphries M, et al Visceral adipose tissue and markers of the insulin resistance syndrome in obese black and white teenagers. Obes Res. 2000;8: 287–93. 10.1038/oby.2000.34 10933304

[25] Gower BA, Nagy TR, Goran MI. Visceral fat, insulin sensitivity, and lipids in prepubertal children. Diabetes. 1999;48: 1515–1521. 10.2337/diabetes.48.8.1515 10426367

[26] Brambilla P, Manzoni P, Agostini G, Beccaria L, Ruotolo G, Sironi S, et al Persisting obesity starting before puberty is associated with stable intraabdominal fat during adolescence. Int J Obes Relat Metab Disord. 1999;23: 299–303. Available: http://www.ncbi.nlm.nih.gov/pubmed/10193876 10193876 10.1038/sj.ijo.0800815

[27] Tong A, Sainsbury P, Craig J. Consolidated criteria for reporting qualitative research (COREQ): a 32-item checklist for interviews and focus groups. Int J Qual Health Care. 2007;19: 349–57. 10.1093/intqhc/mzm042 17872937

